# Is Topiramate Helpful in the Management of Cocaine Use-Related Psychiatric Comorbidities?

**DOI:** 10.7759/cureus.87935

**Published:** 2025-07-14

**Authors:** Thayjas A Patil, Eduardo D Espiridion, Katherine M Napalinga

**Affiliations:** 1 Psychiatry, Drexel University College of Medicine, Philadelphia, USA; 2 Psychiatry, Reading Hospital Tower Health, West Reading, USA

**Keywords:** cocaine abuse, cocaine dependence, cocaine use, cocaine use disorder, large retrospective cohort, psychotherapy, topiramate

## Abstract

Introduction

Cocaine use disorder is a chronic substance use disorder characterized by persistent use of cocaine despite substantial harm and adverse consequences, often developing from patterns of recreational or episodic cocaine use. The pathophysiology of cocaine use is complex, involving the addictive effects on the brain’s limbic system, which affects the region of the brain that regulates pleasure and motivation. In this study, we aimed to examine whether topiramate exposure is associated with the incidence of psychiatric comorbidities in patients with cocaine use.

Methods

This study involved data from 7,325 patients pulled from 66 large healthcare organizations in the TriNetX global health research network and investigated the associations between topiramate exposure and the development of psychiatric comorbidities among patients with cocaine use. The analysis compared patients treated with psychotherapy alone to those treated with psychotherapy and who had topiramate exposure.

Results

Analysis revealed an increased likelihood of developing a depressive episode [odds Ratio (OR) 1.321, risk ratio (RR) 1.145, p<0.0001] and anxiety-related disorders (OR: 1.317, RR: 1.124, p<0.0001) in the topiramate group compared to the group with psychotherapy alone. Although there was a slight increase in suicidal ideation (OR: 1.109, RR: 1.08, p=0.14) and alcohol abuse (OR: 1.005, RR: 1.003, p=0.948) in the topiramate group, these differences were not statistically significant.

Conclusions

These findings suggest that while topiramate may offer some benefits, its exposure is associated with a higher observed incidence of developing depressive and anxiety disorders in patients with cocaine use. On the other hand, topiramate exposure was not significantly associated with increased incidence of developing suicidal ideation or alcohol abuse in this population. Treatment plans should carefully weigh the potential physiological and psychiatric risks when considering topiramate for managing cocaine use, especially in populations already vulnerable to mental health issues.

## Introduction

The 2023 National Survey on Drug Use and Health reported that 1.8% (five million) of individuals aged 12 years or older used cocaine in the past year, with nearly half a million new users. The highest usage was among adults aged 18 to 25 years [1]. This use comes at a cost. Cocaine has been linked to significant healthcare utilization and morbidity [2]. In 2011, over a third of drug-related emergency department visits involved cocaine, and cocaine-related deaths rose from 6,784 in 2015 to 27,569 in 2022 [3,4].

Cocaine use is also frequently comorbid with psychiatric disorders [5-9]. A study of 2,501 lifetime users found that higher lifetime cocaine use was associated with alcohol dependence [adjusted odds ratio (aOR): 1.22], depressive disorders (aOR: 1.07), and generalized anxiety (aOR: 1.12), with prevalence increasing alongside increased use. Near-daily users had a 41.8% risk of lifetime depression, a 13.4% risk of generalized anxiety, and a 66.9% risk of alcohol dependence [10]. Similar results were seen in the relationship between cocaine and suicide. Cocaine use was linked to 18-20% of 749 suicide cases in a New York City study [11]. Another 2021 meta-analysis of 2,252 cocaine users found a prevalence of 43.59% for suicidal ideation and 27.71% for suicide attempts [12].

Cocaine, a central nervous system stimulant, increases energy, alertness, heart rate, body temperature, and blood pressure, contributing to its high addictiveness [13]. However, its continued and habitual use is linked to developing cocaine use disorder [14]. Cocaine blocks the reuptake of dopamine and serotonin, amplifying mesolimbic dopamine signals by increasing both dopamine concentration and duration at nerve terminals [15,16]. The mesolimbic pathway mediates feelings of reward, via projections from the ventral tegmental area (VTA) to the nucleus accumbens, while the mesocortical pathway governs drug craving and compulsive behaviors, via projections from the VTA to the prefrontal, orbitofrontal, and anterior cingulate cortices [17-20]. This neuronal response can be modulated by gamma-aminobutyric acid (GABA), which inhibits dopamine, and glutamate, which increases dopamine. GABAergic medications reduce dopamine response from cocaine administration, while glutamatergic antagonists may reduce cravings and relapse [21-24].

No FDA-approved medications currently exist for treating cocaine use disorder, and abstinence remains the sole endpoint accepted by U.S. regulatory agencies. However, given the challenges for users, especially those with psychiatric comorbidities, there is growing interest in non-abstinent methods [25]. Current interventions include motivational interviewing and group and individual counseling, with cognitive behavioral therapy and voucher-based reinforcement therapy being the most effective [26,27]. A 2019 meta-analysis of 48 RCTs aiming to find potential medication-mediated treatment reported that while antidepressants had no effect, bupropion and topiramate improved abstinence, and antipsychotics improved treatment retention [28].

Topiramate, commonly used for seizure and migraine prevention, is believed to modulate GABA and glutamate neurotransmission by increasing GABA and antagonizing glutamate [29,30]. Blocking glutamate transmission via α-amino-3-hydroxy-5-methyl-4-isoxazolepropionic acid (AMPA) receptor inhibition in the nucleus accumbens has been shown to prevent cocaine self-administration in mice [31]. Other studies have demonstrated that GABAergic medications block cocaine-induced euphoria and also reduce self-administration in animal models [21-23,32,33]. A 13-week double-blind trial found that topiramate increased cocaine abstinence compared to placebo, and another study showed 20% of topiramate-treated patients achieved three weeks of abstinence, versus 6% with placebo at the end of 12 weeks [34,35].

Topiramate also acts as a mood stabilizer, likely through similar GABA-enhancing and glutamate-inhibiting mechanisms [36-38]. Traditional mood stabilizers like lithium are less effective in patients with concurrent substance abuse [39]. Therefore, there is a need for medications that address both cocaine addiction and psychiatric comorbidities, alongside the currently accepted treatment of psychotherapy. To investigate this further, this study aims to evaluate whether topiramate exposure is associated with the incidence of psychiatric comorbidities in patients with cocaine use. We hypothesize that individuals with cocaine use who have had exposure to topiramate will experience lower rates of adverse psychiatric outcomes compared to those unexposed.

## Materials and methods

This retrospective observational cohort study examined the correlation of topiramate exposure on the incidence of psychiatric outcomes in patients with cocaine use, using data from the TriNetX global health research network. TriNetX provides access to EPIC electronic medical records from large healthcare organizations (HCOs). The study analyzed outcomes from 66 HCOs in the US Collaborative Network. This study was exempt from informed consent as it involved secondary analysis of existing data, did not involve intervention or interaction with human subjects, and is de-identified per the de-identification standard defined in Section §164.514(a) of the HIPAA Privacy Rule [40]. TriNetX complies with the ISO 27001:2022 standard and maintains an Information Security Management System (ISMS) to ensure this protection. For statistical analysis, TriNetX utilizes multiple software languages and packages to calculate results, including Apache Commons Math 3.6.1, Hmisc1-1, Survival 3.2-3, Lifelines 0.22.4, Matplotlib 3.5.1, Numpy 1.21.5, Pandas 1.3.5, Scipy 1.7.3, and Statsmodels 0.13.2 in the programming languages of Java 11.0.16, R 4.0.2, and Python 3.7. The latest version of the TriNetX software, as of August 2024, was utilized for the study.

The analysis compared two cohorts: Cohort A (1,332 patients) and Cohort B (6,273 patients). The inclusion criteria for patients in both cohorts consisted of at least one clinical encounter associated with the following diagnostic and procedural terms in their medical record: “Cocaine abuse”(ICD-10-CM code F14.1), “Cocaine dependence” (ICD-10-CM code F14.2), “Cocaine use, unspecified”( ICD-10-CM code F14.9), and “Psychotherapy Services and Procedures” (CPT code 1021137). Cohort A had the additional inclusion criteria of documented evidence of “topiramate” (RxNorm code 38404) exposure. Patients in Cohort B were required to have no record of topiramate exposure to ensure a clear comparison group. Any patients without documentation of the above diagnostic or procedural terms were excluded from the study. The analysis covered patients meeting the criteria up to 20 years ago, including outcomes both before and after the criteria were met. Outcomes analyzed included “Depressive Episode” (ICD-10-CM code F32), “Suicidal Ideations” (ICD-10-CM code R45.851), “Alcohol Abuse” (ICD-10-CM code F10.1), and “Other anxiety disorders” (ICD-10-CM code F41), with each outcome analyzed independently.

Cohort characteristics were analyzed using an independent two-sample t-test for continuous variables and a chi-squared test for categorical variables, assuming unequal variances. Categorical variable distributions were compared between cohorts, and each subcategory was compared to the total of those not in that subcategory but within the associated category.

Outcome data were analyzed using measures of association, comparing the fraction of patients with each selected outcome. Key measures included risk difference, risk ratio (RR), and odds ratio (OR). Kaplan-Meier analysis was employed to estimate outcome probabilities over time, applying censoring for patients who exited the cohort during the analysis. Measures included median survival (days until survival drops below 50%), survival probability at the end of the time window, log-rank test, and hazard ratio (HR).

## Results

Cohort A (with topiramate) included 1,332 patients from 40 providers, and Cohort B (without topiramate) included 6,273 patients from 49 providers. Ten patients from Cohort A and 59 from Cohort B were excluded due to exceeding the 20-year query limit. Final cohort sizes fluctuated due to variations in HCO responses during analysis. Cohort A had 1,300 patients, and Cohort B had 6,082 for depressive episode and suicidal ideation outcome analyses, which were run on Aug 22, 2024, at 18:03:09 UTC and 18:09:51 UTC, respectively. For alcohol abuse and anxiety outcome analyses, Cohort A had 1,311 patients and Cohort B had 6,214 patients, run on Aug 22, 2024, at 18:29:35 UTC and 18:16:02 UTC, respectively. Cohort characteristic analysis is presented in Table 1. 

**Table 1:** Cohort Characteristics for Cohort A and Cohort B Cohort A (with topiramate) and Cohort B (without topiramate). Bold p-values denote statistically significant results using an α<0.05

Cohort Characteristics	Cohort A	Cohort B	P-value
Total patients (Frequency)	1,332	6,273	
Current Age, Years (Average ± Standard Deviation)	49.4 ± 12.5	47.1 ± 14.5	<0.0001
Minimum Age, Years	18	8	
Maximum Age, Years	89	90	
Age at Index, Years (Average ± Standard Deviation)	45.2 ± 12.5	42.3 ± 14.5	<0.0001
Minimum Age, Years	10	3	
Maximum Age, Years	85	90	
Gender			<0.0001
Female (Frequency)	819	2,568	<0.0001
Male (Frequency)	495	3,583	<0.0001
Unknown Gender (Frequency)	18	122	0.177
Ethnicity			0.00194
Not Hispanic or Latino (Frequency)	1,039	5,038	0.0611
Unknown Ethnicity (Frequency)	184	661	0.0007
Hispanic or Latino (Frequency)	109	574	0.2853
Race			0.0033
White (Frequency)	769	3,646	0.6222
Black or African (Frequency)	377	1,823	0.5081
Unknown Race (Frequency)	149	573	0.0284
Other Race (Frequency)	16	134	0.0318
American Indian or Alaska Native (Frequency)	10	23	0.0912
Asian (Frequency)	10	55	0.7566
Native Hawaiian or Other Pacific Islander (Frequency)	10	19	0.0319

Cohort characteristics analysis revealed significant differences between cohorts in current age (p<0.0001) and age at index (p<0.0001). Significant differences were also observed in gender (p<0.0001), ethnicity (p=0.00194), and race (p=0.0033) when examining overall category distributions. However, individual subcategories, including “Unknown Gender,” "Not Hispanic or Latino," "Hispanic or Latino," "White," "Black or African," "American Indian or Alaska Native," and "Asian," were not significantly different. Cohorts were then analyzed for the presence and likelihood of outcome measures (Table 2 and Table 3).

**Table 2:** Patient Outcomes by Cohort Cohort A (with topiramate) and Cohort B (without topiramate)

Outcome	Cohort	Patients in Cohort (Frequency)	Patients With Outcome (Frequency)	Risk
Depressive Episode	A	1,300	714	0.549
	B	6,082	2,918	0.48
Suicidal Ideations	A	1,300	339	0.261
	B	6,082	1,468	0.241
Alcohol Abuse	A	1,311	348	0.265
	B	6,214	1,644	0.265
Other Anxiety Disorders	A	1,311	797	0.608
	B	6,214	3,360	0.541

**Table 3:** Measure of Association Analysis by Outcome Bold p-values denote statistically significant results using an α<0.05 CI: Confidence Interval; Z: Z-Score

Outcome	Risk Difference	95% CI	Z	P-value	Risk Ratio	95% CI	Odds Ratio	95% CI
Depressive Episode	6.90%	(4.0%, 9.9%)	4.547	<0.0001	1.145	(1.083, 1.210)	1.321	(1.171, 1.490)
Suicidal Ideations	1.90%	(-0.7%, 4.6%)	1.477	0.14	1.08	(0.976, 1.196)	1.109	(0.967, 1.272)
Alcohol Abuse	0.10%	(-2.5%, 2.7%)	0.066	0.948	1.003	(0.909, 1.108)	1.005	(0.878, 1.150)
Other Anxiety Disorders	6.70%	(3.8%, 9.6%)	4.448	<0.0001	1.124	(1.070, 1.181)	1.317	(1.166, 1.487)

The topiramate group had a 6.90% increased risk of developing a depressive episode (p<0.0001) and a 6.70% higher risk for anxiety-related disorders (p<0.0001). The risk of suicidal ideation was slightly higher but not significant (p=0.14), and alcohol abuse risk similarly showed a slight but insignificant difference (p=0.948). The risk ratio revealed how likely topiramate exposure was to predict the development of an outcome, while the odds ratio indicated how likely a patient with the outcome had been exposed to topiramate in the past. A risk ratio of 1.145 and an odds ratio of 1.321 indicated a significant association between topiramate exposure and depressive episodes, and a risk ratio of 1.24 and an odds ratio of 1.317 showed a similar association with anxiety-related disorders. On the other hand, the risk ratios for suicidal ideation (1.08) and alcohol abuse (1.003) and the odds ratios for suicidal ideation (1.109) and alcohol abuse (1.005) were not significant, indicating no increased likelihood of these outcomes with topiramate exposure compared to psychotherapy alone. To better understand the temporality of outcome measures development throughout the study period, survival analysis, log-rank test, and hazard ratio analysis were conducted, as shown in Table 4, Table 5, and Table 6, respectively.

**Table 4:** Survival Analysis Cohort A (with topiramate) and Cohort B (without topiramate)

Outcome	Cohort	Median Survival (Days)	Survival Probability at End of Time Window
Depressive Episode	A	489	12.51%
	B	885	23.72%
Suicidal Ideations	A	N/A	60.91%
	B	N/A	54.79%
Alcohol Abuse	A	N/A	61.26%
	B	N/A	50.06%
Other Anxiety Disorders	A	287	10.65%
	B	486	0.00%

**Table 5:** Log-Rank Test Bold p-values denote statistically significant results using an α<0.05 X^2^: Chi-Square; df: Degrees of Freedom

Outcome	X^2^	df	P-value
Depressive Episode	23.626	1	<0.0001
Suicidal Ideations	3.037	1	0.081
Alcohol Abuse	0.049	1	0.825
Other Anxiety Disorders	28.105	1	<0.0001

**Table 6:** Hazard Ratio Analysis Bold p-values denote statistically significant results using an α<0.05 CI: Confidence Interval; X^2^: Chi-Square; df: Degrees of Freedom

Outcome	Hazard Ratio	95% CI	X^2^	df	P-value
Depressive Episode	1.224	(1.128, 1.329)	8.034	1	0.005
Suicidal Ideation	1.111	(0.987, 1.250)	0.127	1	0.721
Alcohol Abuse	1.013	(0.902, 1.137)	0.081	1	0.776
Other Anxiety Disorders	1.232	(1.140, 1.331)	2.042	1	0.153

The survival analysis indicated that, by the end of the 20-year period, the likelihood of not developing a psychiatric comorbidity was higher in the topiramate group for all outcomes except depressive episodes, which were 11.21% less likely in the non-topiramate group. The log-rank test showed significant differences in survival distributions in the time to outcome for depression (X²: 23.626, p<0.0001) and anxiety (X²: 28.105, p<0.0001). The hazard ratio revealed a 22.4% higher risk of developing a depressive episode (p=0.005) and a 23.2% higher risk of anxiety-related disorders (p=0.153) in the topiramate group at any point in time. Suicidal ideation and alcohol abuse showed no significant differences in survival distributions, with a hazard ratio indicating an 11.1% increased risk for suicidal ideation (p=0.721) and a 1.3% increase for alcohol abuse (p=0.776).

## Discussion

While initially approved by the FDA for epilepsy and migraines, topiramate was later adopted by psychiatrists as a mood stabilizer. Its off-label uses expanded as clinicians continued to discover new applications for the medication. Over time, growing evidence has demonstrated topiramate’s potential effectiveness in treating mood, anxiety, eating, personality, and substance use disorders [10,41,42].

Depressive episodes

Our results showed a significantly increased risk of developing a depressive episode in patients with cocaine use who had topiramate exposure, compared to psychotherapy alone. This association raises concern that topiramate may be correlated with increased mood disorder development in this vulnerable population. No specific studies were found on preventing major depressive disorder (MDD) with topiramate in patients who use cocaine, and evidence on its impact in treating MDD is mixed.

Some studies support topiramate’s use in treating MDD. A double-blind trial of 64 women with recurrent MDD found that 200 mg of topiramate daily for 10 weeks significantly reduced Hamilton Depression Rating scores (HDRS) [43]. Another study found that topiramate produced a 50% reduction in HDRS-17 scores in 56% of bipolar depression patients [44]. In a study involving 83 treatment-refractory bipolar depression patients, adjunctive topiramate greatly improved HDRS-17 scores (score at endpoint 0-5) in 54% of patients [45]. An open-label study found that 44% of treatment-resistant MDD patients responded to topiramate after 17.7 weeks, and another eight-week trial in treatment-resistant MDD showed a 32% reduction in HDRS scores compared to 5.5% with placebo [46,47].

However, there is also evidence against topiramate use in MDD. A case study found that 300 mg of topiramate over eight weeks lacked efficacy in unipolar depression, with anxiety and depressive symptoms leading to discontinuation [48]. Topiramate has also been linked to depression in 10% of patients, and the same has been demonstrated in healthy volunteers [49,50]. This relationship was later shown to be dose-dependent and increased with psychiatric history [51].

Our findings suggest that while topiramate may help treat depressive disorders, particularly in treatment-resistant cases, it may also be associated with an increased incidence of depressive mood disorder development. Risk factors, such as concurrent cocaine abuse, may contribute to this effect.

Suicidal ideation

Our results indicated a slight increase in suicidal ideation in patients with cocaine use treated with psychotherapy and had topiramate exposure, but this risk was not significantly different from psychotherapy alone, suggesting topiramate is not strongly associated with the development of suicidal ideation in these patients. This contrasts with existing evidence linking topiramate and antiepileptics to increased suicidality. In 2008, the FDA warned that all antiepileptics may increase the risk of suicidal ideation, attempts, and completion [52]. Though debated, several studies support this finding, particularly in patients with preexisting psychiatric conditions [53-55]. In patients with newly diagnosed bipolar disorder, some studies have reported a significant increase in suicide attempts with topiramate exposure, though the magnitude of risk varies between studies [56-58].

No data was found on the effectiveness of topiramate specifically for preventing suicidal ideation. One possible explanation for reduced suicide risk is improved mood stability, as seen in the decreased suicidality in bipolar patients on topiramate [59]. As shown, these findings are mixed, and it is unclear whether this stability played a role in our results for patients with cocaine use.

Alcohol abuse

The associated risk of developing alcohol abuse was slightly higher in the group treated with psychotherapy and had topiramate exposure, but the difference was not significant compared to psychotherapy alone. This suggests topiramate exposure was not associated with increased incidence of alcohol abuse in these patients. Although no studies were found on topiramate preventing or inducing alcohol use disorder, considerable evidence supports its use in treating alcohol abuse.

Several studies have demonstrated topiramate's effectiveness in treating alcohol abuse. A double-blind study of 150 individuals with alcohol dependency found that 25-300 mg of topiramate daily for 12 weeks reduced alcohol consumption by 2.88 drinks per day, 27.6% fewer heavy drinking days, and 26.2% more abstinent days compared to placebo, also demonstrating fewer drinking obsessions and automaticity [60]. A similar study with 371 participants confirmed significant reductions in heavy drinking days, with other studies replicating these results [61-65]. Studies with low-dose topiramate (75-100 mg/day) also showed reduced cravings, withdrawal symptoms, and relapse rates [66,67].

However, some evidence contradicts these findings. In 2013, one trial of 170 cocaine- and alcohol-dependent patients found no reduction in alcohol use with topiramate, and another trial of 106 alcohol-dependent patients reported no significant difference between placebo and topiramate [68,69].

Despite contradictory evidence, non-abstinence goals may still reduce the risk of clinically important outcomes [10]. Overall, our evidence supports that topiramate was not associated with increased risk of alcohol abuse in patients with cocaine use, aligning with some of the compulsion-limiting evidence in the literature.

Other anxiety disorders

As a stimulant, cocaine poses a significant risk for anxiety-related disorders. Although there is neurobiological potential for topiramate to help treat cocaine use, our data suggest that it may be associated with increased risk of anxiety. Patients with cocaine use treated with psychotherapy and who had topiramate exposure showed a significantly higher risk of developing anxiety-related disorders compared to psychotherapy alone. Limited data exists on topiramate's role in preventing anxiety, and its effects on treating anxiety-related disorders are mixed.

Many studies cite anxiety as a common reason for discontinuing topiramate [42]. One case report linked topiramate to new-onset panic attacks, which ceased after stopping the drug but resumed when the patient restarted it [70]. However, other studies have demonstrated topiramate's anxiolytic properties. In an eight-week randomized trial, topiramate significantly reduced agitation, psychological anxiety, and somatic anxiety in treatment-resistant depression patients [46]. In both animal studies and trials in alcohol-dependent patients, topiramate caused significant reductions in anxiety [67,71]. A similar finding was observed in patients with post-traumatic stress disorder (49% symptom reduction) and social phobia (45% symptom reduction) [72,73]. Despite data supporting topiramate’s anxiolytic effects, our findings indicate an association with increased anxiety diagnoses, particularly in patients with cocaine use, where the stimulant effects of cocaine may already exacerbate anxiety.

Limitations

While the TriNetX platform provided a robust dataset, it has certain limitations. The platform, based on EPIC medical records since 2006, had unidentifiable, non-responding HCOs, leading to minor variations in cohort sizes and characteristics based on time of analysis. Among cohort characteristics, significant differences were found, but their impact on outcomes is unclear due to the lack of raw data available for analysis. Nevertheless, the process for selecting cohorts was applied consistently and automatically across groups using the appropriate inclusion and exclusion criteria. Having U.S. data from large HCOs also limits generalizability to smaller clinics or international populations.

The lack of access to raw data, including information on patient dropouts, comorbidities, and socioeconomic status, also limited our ability to assess the impact of other potential confounding variables on the results. It was unclear if topiramate was used specifically for cocaine use or other conditions, which may independently affect the risk of outcomes. For example, suicide risk is associated with primary mood disorders [74,75]. The highest predictor of suicide is prior attempt, with one study suggesting up to 30% risk of reattempt within eight years [76]. Patients prescribed topiramate may also have had more severe psychiatric symptoms to begin with. Without accounting for comorbidities or other potentially psychotropic medications, it is difficult to assess whether topiramate exposure is independently associated with these outcomes. 

Because patients were included based on meeting criteria at any point over the past 20 years, with outcomes occurring before or after that point, temporal ordering between exposure and outcomes is limited. However, given that individuals with cocaine use disorder often engage in cocaine use well before it is formally documented in clinical records, this lack of precise temporality may be less consequential for interpreting associations related to cocaine exposure.

Limited access to raw data also affected survival probabilities for anxiety-related disorders. Analysis errors, such as the zero-survival probability at the end of the time window, were likely due to platform issues, given that not all patients experienced that outcome. Additionally, the dosage and frequency of topiramate exposure were unknown, which is concerning given the dose-dependent relationship with psychiatric comorbidities [51]. Cocaine use severity and active use status were also not specified, as this also has a dose-dependent relationship with psychiatric comorbidities [10]. Despite these limitations, the data offers useful insights into the associations between topiramate exposure and mental health outcomes across cohorts, regardless of other conditions and circumstances.

## Conclusions

This study aimed to evaluate whether topiramate exposure is associated with the incidence of psychiatric comorbidities in patients with cocaine use. The results indicate that while topiramate exposure is not significantly associated with the risk of developing suicidal ideation or alcohol abuse compared to psychotherapy alone, it is correlated with higher rates of developing depressive episodes and anxiety-related disorders. These findings highlight the complexity of using topiramate in patients who use cocaine, given the preexisting vulnerabilities in this population. Although literature supports topiramate’s use in conditions like epilepsy and alcohol dependence, the increased likelihood of depressive episodes and anxiety raises concerns that topiramate exposure may be associated with worsening psychiatric comorbidities in these patients, necessitating a more nuanced approach to its prescription. Given the study's limitations, such as the lack of controlling for topiramate dosage, comorbidities, and cocaine use severity, further research is needed to fully understand topiramate's impact in this population. Future studies should focus on clarifying the risk-benefit profile of topiramate in treating cocaine use, especially in patients with or at risk of comorbid psychiatric conditions, to optimize treatment strategies and improve patient outcomes.
